# Commentary on “Immler et al. (2021) The voltage‑gated potassium channel Kv1.3 regulates neutrophil recruitment during inflammation” Cardiovasc Res 2021 (doi:10.1093/cvr/cvab133): Roll over—Kv1.3 ! The function of a voltage-gated potassium channel in leucocytes revealed

**DOI:** 10.1007/s00424-021-02615-1

**Published:** 2021-08-30

**Authors:** Jochen Roeper

**Affiliations:** Institute for Neurophysiology, Neuroscience Center, Theodor-Stern-Kai 7, 60590 Frankfurt, Germany

At least from an electrophysiological vantage point, you might be forgiven for wondering what exactly voltage-gated potassium (Kv) channels might do in leucocytes. They lack the fast and furious 100 mV membrane potential swings during action potential (AP) firing found in excitable cells, where Kv channels opening helps to quickly repolarize the initial upstroke of the action potential mediated by even faster voltage-gated sodium channels. Moreover, in presynaptic terminals, Kv channels limit the calcium influx via voltage-gated calcium channels and in turn dampen calcium-dependent exocytosis of synaptic vesicles. In essence, we are familiar with Kv channels as powerful players enacting negative feedback on voltage-dependent calcium signaling. However, there is an equally important, very different choreography between Kv channels and calcium signaling in non-excitable cells. This pas-de-deux is more subtle, slower as well as synergistic with Kv channel opening stabilizing negative membrane potentials with millivolt precision and thereby enhance the driving force for calcium influx through voltage-independent store-operated calcium channels (CRAC, ORAI-STIM) [9]. This alternative mechanism of supportive Kv-calcium interaction and its important functional downstream consequences were elegantly worked out by Cahalan and colleagues for the adaptive immune system in showing by now classical work that a particular isoform (Kv1.3) of Kv1 (“shaker”) channel family plays this crucial role in the context of T-cell activation [1, 2]. As one of the translational consequences of their work, selective Kv1.3 channel inhibition has been identified as an attractive target for immunomodulation [3], not only in T-cells but also in microglia in the brain [4].

Immler and colleagues [5] now close an important gap in our knowledge. They convincingly demonstrate that Kv1.3 channels also possess an essential role for enabling functionally relevant calcium signaling in the innate immune system. In particular, they provide experimental evidence that Kv1.3 channels are not only functionally expressed in human neutrophil granulocytes, but their selective pharmacological inhibition (by PAP-1) or genetic deletion (Kv1.3 knockout mice) impairs sustained calcium influx and in turn several neutrophil key functions in acute inflammation. One of the beauties of this study lies in the breath of functional validation of these Kv1.3 downstream processes. The authors showed that Kv1.3 function is relevant for crawling under flow conditions, post-arrest cellular modifications (like, e.g., paxilin phosphorylation), stable attachment as well as transmigration, and even phagocytosis [5]. Importantly, they also demonstrated these Kv1.3 neutrophil functions in an in vivo inflammation model by intravital microscopy of post-capillary venules of an inflamed muscle. Finally, they confirmed Kv1.3 function for neutrophil extravasation in yet another in vivo model of TNF-alpha induced peritonitis [5].

Let us return to Kv1.3 for a closer look. What functional features single out this Kv1 isoform—among the vast diversity of Kv channels [6, 7] with 10 genes coding for Kv1 alpha subunits alone for enhancing and shaping the calcium influx in leukocytes that orchestrates many of their cellular key functions. In addition to a fine-tuned voltage dependence of activation, it might be its exceptionally slow C-type inactivation [8] that renders this isoform also a time-keeper of calcium signaling. Now that Immler and colleagues [5] firmly established Kv1.3 function in neutrophils per se the next generation of studies can explore the fine tuning of Kv1.3 function in these cells including its functional regulation by auxiliary beta-subunits and other protein partners [6] as well as the control of expression and dynamic membrane delivery [2] of this exciting ion channel complex.
